# Psychiatric Morbidity and Diagnostic Latency among Hungarian Juvenile Offenders

**DOI:** 10.1192/j.eurpsy.2026.11292

**Published:** 2026-07-31

**Authors:** M. Z. Bellavics, N. E. Baráth, J. Haller

**Affiliations:** 1 Faculty of Law Enforcement, Department of Behavioural Sciences and Criminal Psychology; 2 István Nemeskürty Faculty of Teacher Training, Department of Teacher Training, University of Public Service, Budapest, Hungary

## Abstract

**Introduction:**

The high prevalence of psychiatric disorders among juvenile offenders has been well documented (Odgers *et al.*, Can Child Adolesc Psychiatr Rev 2005; 14:26–29). Although most detention facilities conduct an initial medical screening as part of standard intake procedures, substantial evidence indicates that a significant proportion of mental disorders remain undetected. (Mitchell *et al.*, J Forensic Psychiatry Psychol 2011; 22:381–394). Consequently, despite extensive research efforts, the true extent and nature of psychiatric morbidity among juvenile offenders remain a controversial.

**Objectives:**

The aim of the present study was to examine the mental health status of juvenile offenders in Hungary. The study design included both cross-sectional and retrospective approaches.

**Methods:**

The research sample consisted of 103 male juvenile offenders with a mean age of 16.4 years (SD = 1.317). The Mini International Neuropsychiatric Interview for Children and Adolescents (MINI-KID; Sheehan et al., J Clin Psychiatry 2010; 71:3013–326) was administered. In addition, a brief interview focusing on medical history, along with a retrospective analysis of medical records, was conducted.

**Results:**

Based on clinical records, the prevalence of psychiatric disorders was 34% (N = 35). During the interviews, 61.5% (N = 64) of the participants reported at least one prior mental health intervention. According to the MINI-KID assessment, 98.1% (N = 101) of the juvenile offenders met the diagnostic criteria for at least one mental disorder. The mean number of comorbid psychiatric disorders identified by the MINI-KID was 7.07 (SD = 3.448). As shown in Figure 1, the rate of psychiatric morbidity varied substantially depending on the assessment method.

**Image 1:**
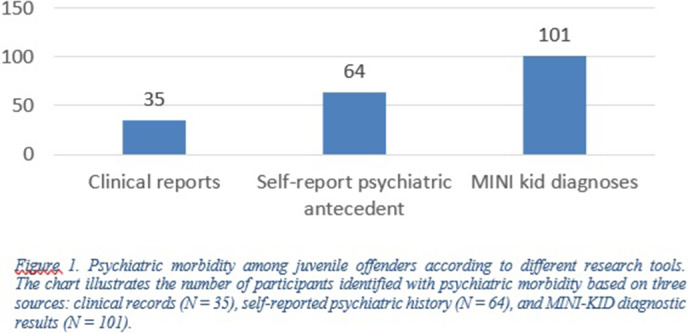

**Conclusions:**

The results of the present study demonstrate a high level of psychiatric morbidity and diagnostic latency among Hungarian juvenile offenders. These findings highlight the importance of conducting comprehensive psychiatric evaluations that go beyond clinical records and self-reported information. The incorporation of structured screening tools into routine mental health assessments in juvenile correctional facilities should be strongly considered.

**Disclosure of Interest:**

None Declared

